# Impact of Heat Wave Definitions on the Added Effect of Heat Waves on Cardiovascular Mortality in Beijing, China

**DOI:** 10.3390/ijerph13090933

**Published:** 2016-09-21

**Authors:** Wentan Dong, Qiang Zeng, Yue Ma, Guoxing Li, Xiaochuan Pan

**Affiliations:** 1 Department of Occupational and Environmental Health, School of Public Health, Peking University, No. 38, Xueyuan Rd, Haidian District, Beijing 100191, China; zhenshi365@126.com (W.D.); xcpan@bjmu.edu.cn (X.P.); 2 Tianjin Center for Disease Control and Prevention, Tianjin 300011, China; zengqianghaiyan@126.com; 3 Department of psychology, DePauw University, 408 South Locust Street, Greencastle, IN 46135, USA; mayue601@163.com

**Keywords:** heat waves, added effect, different definitions, cardiovascular mortality, modification

## Abstract

Heat waves are associated with increased mortality, however, few studies have examined the added effect of heat waves. Moreover, there is limited evidence for the influence of different heat wave definitions (HWs) on cardiovascular mortality in Beijing, the capital of China. The aim of this study was to find the best HW definitions for cardiovascular mortality, and we examined the effect modification by an individual characteristic on cardiovascular mortality in Beijing, a typical northern city in China. We applied a Poisson generalized additive approach to estimate the differences in cardiovascular mortality during heat waves (using 12 HWs) compared with non-heat-wave days in Beijing from 2006 to 2009. We also validated the model fit by checking the residuals to ensure that the autocorrelation was successfully removed. In addition, the effect modifications by individual characteristics were explored in different HWs. Our results showed that the associations between heat waves and cardiovascular mortality differed from different HWs. HWs using the 93th percentile of the daily average temperature (27.7 °C) and a duration ≥5 days had the greatest risk, with an increase of 18% (95% confidence interval (CI): 6%, 31%) in the overall population, 24% (95% CI: 10%, 39%) in an older group (ages ≥65 years), and 22% (95% CI: 3%, 44%) in a female group. The added effect of heat waves was apparent after 5 consecutive heat wave days for the overall population and the older group. Females and the elderly were at higher risk than males and younger subjects (ages <65 years). Our findings suggest that heat wave definitions play a significant role in the relationship between heat wave and cardiovascular mortality. Using a suitable definition may have implications for designing local heat early warning systems and protecting the susceptible populations during heat waves.

## 1. Introduction

Due to global climate change and the urban heat island effect, heat waves have become frequent extreme weather events worldwide. Especially in recent decades, heat waves have been increasing in intensity, duration, and frequency [1,2]. Thus, a discussion on the impact of heat waves on human health problems was inevitable. The uniform effect of heat waves on mortality was assessed in many epidemiological studies across various regions [3,4,5,6,7,8]. However, those previous studies were focused on the independent effect of a daily high temperature, and studies about the added effect due to the duration of sustained heat for several consecutive days are still rare in Asia. In America, the added wave effect was small (0.2%–2.8% excess relative risk) [9]. However, in Asian cities, the added effect of heat waves was large (5.8%–18.1%) [6]. Therefore, the existence and size of the added effects are still vague and more research is needed on the impact of this added effect on human health problems, ignoring the independent effect of extreme temperature.

There is no standard definition for heat waves [10,11], and therefore, heat wave definitions (HWs) vary in the metrics and threshold of the temperature and in the duration. Recent studies have indicated that the variety of available HWs has led to a substantial divergence in the evaluation of health risk, giving rise to confusion when selecting an appropriate definition for its application in an intervention study [12,13]. Therefore, to identify the most appropriate definition for heat wave warning systems, further research is needed to use various definitions for heat waves and data over a long duration to determine the impact of heat waves on mortality.

Studies have shown that the effects of heat waves on mortality vary significantly by the type of disease and that heat waves have a greater influence on cardiovascular mortality than they have on respiratory and total mortality [14,15], which might be due to the impaired cardiac function and impaired vasodilation function that contribute to increased cardiovascular mortality. Within these various types of diseases, cardiovascular diseases have been considered the prime killers over the past decade and have occupied a great proportion of the total disease burden [16]. Hence, studies on cardiovascular mortality are necessary for creating heat wave warning systems to reduce the associated adverse health impacts and to prevent residents from dying in future heat waves. Furthermore, the influence of heat waves on mortality can be modified by individual characteristics, including gender, age and social economic conditions [17,18,19]. Thus, different heat wave definitions, based on duration, may influence the modifying effect of individual characteristics on the estimates of mortality.

In this study, we aimed to examine the influence of different heat wave definitions on the added effects of heat waves on cardiovascular mortality and explored whether the effect modifications by individual characteristics changed under different heat wave definitions in Beijing, a city with a temperate continental monsoon climate, to determine the most proper definition for heat wave warning systems and prepare individuals for future heat waves.

## 2. Materials and Methods

### 2.1. Study Area

The study area is focused on Beijing, which is the capital and the second largest city in China. Beijing has a population of over 17 million, according to the 2009 statistics. As a typical northern city with a latitude of 39′′26′–41’’03′ N, Beijing has a temperate continental climate. The climate in Beijing is a quite dry, monsoon-influenced continental climate with four distinct seasons, including hot rainy summers and cold windy winters. The eight districts in the urban area of Beijing were chosen as the study areas.

### 2.2. Data Collection

The data for the primary causes of death were collected from the Center for Public Health Surveillance and Information Service of Chinese Center for Disease Control and Prevention (China CDC). The data collected include major reasons of death from the beginning of 2006 to the end of 2009. The flow of the death data was collected as follows. First, each death certificate was filled out by the health service centers and hospitals with the primary cause of death. Then, the officials would check and confirm the cause of death and report it to the Chinese CDC through the system. The Chinese CDC has archived all death certificates in electronic form since 2004 for the entire country. The data are updated every year by the Chinese CDC. Therefore, the data on the primary cause of death represent the mortality rate of each city. In addition, the data include information on the death date, address, age, gender and cause of death. The causes of deaths were classified according to the International Classification of Diseases, 10th Revision (ICD-10). I00–I99 is the corresponding ICD-10 code for cardiovascular disease, including hypertensive disease (I10–I15), ischemic heart disease (I20–I25), cerebrovascular disease (I60–I69) and other diseases of the circulatory system. This study was approved by the Ethics Committee of Peking University Health Science Centre, Beijing, China.

The daily meteorological data, including the relative humidity (RH) and the mean temperature (MT) during 2006–2009, were obtained from the China Meteorological Data Sharing Service System with stations located in the urban districts of Beijing. In addition, the daily ambient concentrations of PM_10_ for the same period were retrieved from the local Environmental Monitoring Center, which has 12 stations distributed throughout Beijing. Further, the daily arithmetic mean concentrations of particles with an aerodynamic diameter less than 10 microns (PM_10_) were collected from monitoring stations in Beijing.

### 2.3. Heat Wave Definitions

In the absence of a single, global heat wave definition, two types of heat wave definitions are generally used. One heat wave definition uses a temperature higher than an absolute threshold over consecutive days, and the other uses is based on a relative threshold over consecutive days [20,21]. In this study, the heat wave definition based on a relative threshold was used, and research was conducted on the local long-term weather and the acclimatization to local weather types. Twelve definitions were used in the relative average temperature threshold (90th, 93th, and 95th) and a duration of more than 2, 3, 4 and 5 days (Table 1). We did not include the 97th percentile because only a few days of heat waves were found in that study period.

### 2.4. Statistical Analysis

To explore the effects of heat waves on cardiovascular mortality, we applied a Poisson generalized additive approach. A heat wave was defined as the mean temperature above a temperature threshold (i.e., 90th, 93th, and 95th) for 2, 3, 4 or 5 consecutive days in the summer season (from 1 June to the end of August). A dummy heat wave variable (0 or 1) was used for each day (e.g., using 1 for the heat wave days indicated that the daily mean temperature was equal to or higher than the temperature threshold for 2, 3, 4 or 5 days, and 0 was used for non-heat wave days):

Log(*Yt*) = α + *ns* (doy, 3 × 4) + *ns* (year, 3) + λTt, l + *ns* (*RHt*, 3) + η*DOWt* + κ*PMt* + *HW* (Model 1)

where *t* is the day of the observation and *Yt* is the observed daily death number at day *t*, which was assumed to follow a Poisson distribution. α is the intercept and *ns* represents the natural spline function. The ‘year’ and ‘DOW’ terms describe a regular seasonal trend and a long-term trend, using 3 and 3 degrees for freedom (*df*), respectively. Tt, l is a two-dimensional B spline for temperature and the lag days, λ is the vector of the coefficients for Tt, l and l is the lag days. A B spline with five *df* was used for temperature, with four *df* for the lag days (≤7 days) considering the hot effects lasting within one week [22,23]. *RHt* is the daily level for relative humidity. *DOWt* is day of the week on day *t* and η is the vector of the coefficients. *PMt* is the daily level for PM_10_ and κ is the vector of the coefficients. *HW* is a dummy variable. To estimate the effects of HWs on cardiovascular mortality, we adopted 12 different HWs in model 1. In addition, we validated the model fit by checking the residuals to ensure that autocorrelation had been successfully removed.

We also used a second approach to characterize and to capture the characteristics of the added effects (above the 95th percentile threshold). In model 1, the variable (*HW*) was replaced by *f(HW)*. The function *f*, denoting the added effects in terms of consecutive heat wave days *d*, is specified in two ways as follows: through a step function (strata breaks: 1, 2, and 5) or through quadratic splines with 4 *df* (2 knots at 2 and 5 days).

To explore the modifying effect of individual characteristics, such as gender and age, we also conducted a similar study in different population groups. Sensitivity analyses were conducted to check the robustness of the results. We changed the *df* for humidity (2 and 4 *df*), for the day of the summer season (2 and 4 *df*), and for removing air pollution and then assessed the robustness of the modeling outcomes.

## 3. Results

Table 1 shows the number of heat wave days during 2006–2009 in Beijing under different heat wave definitions. As expected, less heat wave days were observed as the relative threshold of the daily mean temperature increased from the 90th to the 95th percentile (e.g., HW1, HW5, and HW9). In addition, the duration of the heat waves increased from two consecutive days to five consecutive days (e.g., HW1–HW4). The longest duration of heat waves also varied under different heat wave definitions, and the change trend was similar to the number of heat wave days. When the definition (HW1) was ≥90th percentile and ≥2 consecutive days, the longest duration of heat waves was a maximum of 15 days and then decreased as the threshold of the daily mean temperature and the consecutive days of heat waves increased. The longest duration of HW12 (≥95th percentile and ≥5 consecutive days) was the minimum quantity of 8 days.

Table 2 summarizes the characteristics of the population, mortality and the climatic variables in Beijing. The daily mean temperature was 13.6 °C (range from −10.1 to 31.6 °C) during the study period in 2006–2009. The daily average count of cardiovascular deaths was 47 in Beijing; of this total, an average of 8 and 39 people died daily in the young and old groups, respectively, during the study period in 2006–2009. The number of deaths in the old group (83%) was higher than that in the young group (17%). The daily average male count of cardiovascular mortality was 26 people, which was slightly higher than the female count (21 people).

Table 3 shows the added effect of heat waves on cardiovascular mortality depending on the different definitions of the model. For the corresponding graphs, see Figure S4. The effect was estimated as the relative risk. We observed that a total of four HWs (HW7, HW8, HW11 and HW12) were statistically significantly associated with the total, old, and female cardiovascular mortality. Of all of the 12 HWs, HW8 was associated with the highest mortality risk with an 18% increase in total cardiovascular mortality, a 24% increase in old cardiovascular mortality, and a 22% increase in female cardiovascular mortality. However, not all of the 12 HWs were statistically significantly associated with the adult, young and male groups. When heat wave was defined with the threshold value of the 93th percentile, the heat waves added an additional impact to the effect of the high temperature and caused a higher mortality rate than did the other temperature thresholds (90th and 95th percentiles). The magnitude of the added effect on the older group’s mortality was greater than that found for the younger group, and the added effect on female mortality was higher than that of the total and the male group. In the model without adjusting for PM_10_ in Table S2, the added effect of the heat waves changed slightly, and the statistical significance of the added effects remained uniform. For example, the estimated heat wave effects in HW8 on cardiovascular mortality among the elderly were consistent after adjusting for PM_10_ (1.24%, 95% confidence interval (CI): 1.10%, 1.39%) and before adjusting for PM_10_ (1.23%, 95% CI: 1.10%, 1.38%).

**Figure 1:**
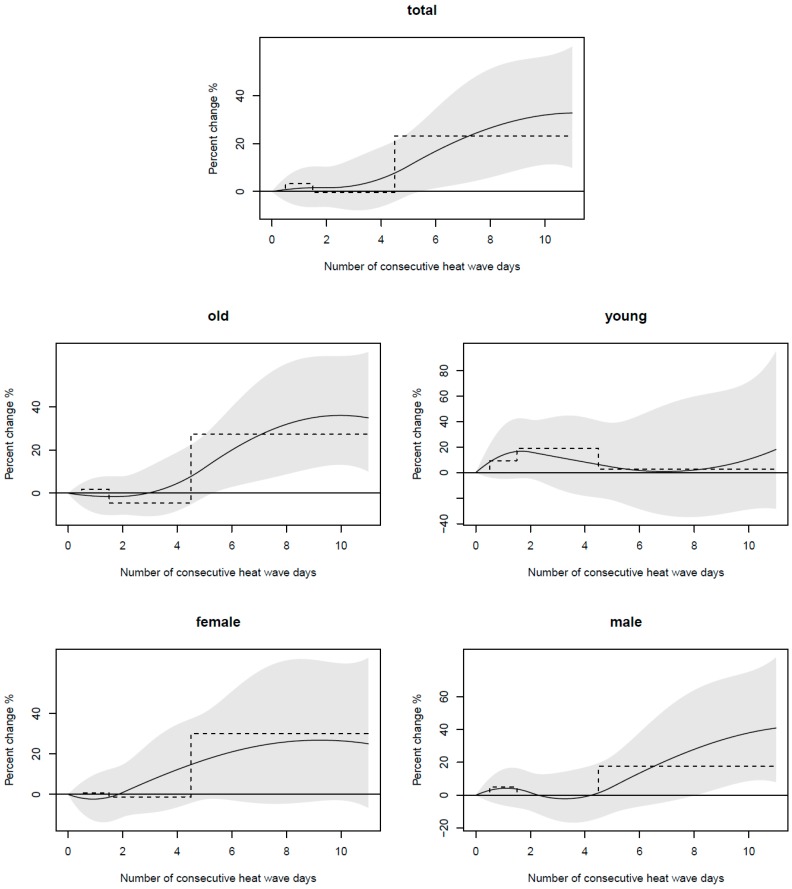
Average wave effect of consecutive heat wave days (above the 93th percentile), as estimated by quadratic spline (continuous line), with a 95% confidence interval (CI) (grey area), and by a step function (dashed line).

The added effect, which was modeled simultaneously by both a step function and a quadratic spline and elaborated by an increased risk for consecutive heat wave days, is described in Figure 1.

In the total group, the analysis shows an increasing trend when the heat was sustained. We observed that it brought some risks after five days of uninterrupted heat, and the relative risks are statistically significant among the total and the older group. However, the heat wave effects appeared after eight consistent days in the male group, and no significant effects were observed in the female group, which was different from the results in Table 3, and further study is needed to check the robustness.

**Figure 2:**
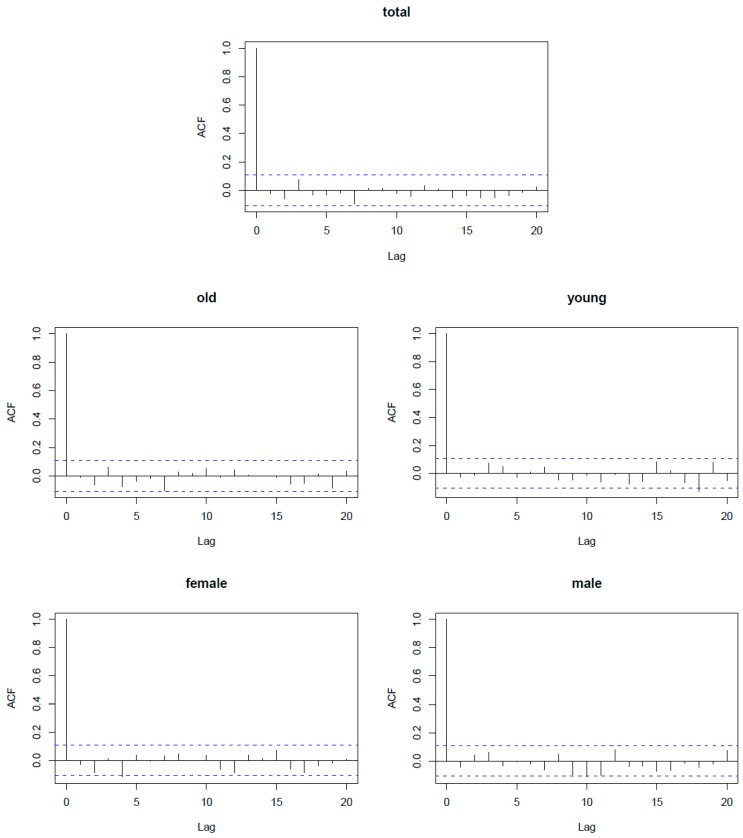
The residual autocorrelation figure (ACF) of the model in the definition of ≥5 consecutive days.

Our sensitivity analysis showed that the estimated mortality risks were robust using different degrees of freedom (2–4) for time trends and degrees of freedom (2–4) for the relative humidity (see Table S1). In the model of the residual autocorrelation function test (Figure 2), we observed that almost all the heights of the estimated values were under the dotted lines from lag 1 to lag 20, indicating that our models are highly stable and that the probability of the estimated values is reliable.

## 4. Discussion

In this study, we found that HW8 best captured the added effects of heat waves on cardiovascular mortality in Beijing. The characteristic of the added effects of heat waves on cardiovascular mortality differed by age and gender. The elderly were more sensitive than the young, and females were more sensitive than males. We also found that heat waves with a longer duration increased the risks of cardiovascular mortality more than those with shorter duration among all of the groups, and five consecutive days of heat waves had significant effects on cardiovascular mortality in the total and old groups.

We compared 12 heat wave definitions and found that using a consecutive duration ≥5 days in ≥93th percentile of temperature was the best way to describe the added effects of heat wave on cardiovascular mortality. The heat wave definitions that used a duration ≥2 days included 12 types of heat waves that could last for more than two days. However, the heat wave definitions that used a duration ≥5 days, excluding those heat waves with a duration of 2–4 days, indicated that a heat wave definition with a duration ≥5 days might have an added effect of heat waves on cardiovascular mortality. However, the heat wave definition that used a shorter duration did not have added effects. This finding is very important and useful because it can help the local government make efficient policies to protect people from prolonged periods of extreme temperatures. By comparing the effects of different relative temperature thresholds, we found that the effects of the heat wave definitions based on an average temperature threshold of the 93th and 95th percentile were greater than the effects of those based on an average temperature threshold of the 90th percentile under different durations (3, 4, and 5 days) on total cardiovascular mortality. This is similar to recent studies in Asia, in which the heat wave definitions based on an average temperature threshold of the 95th percentile were higher than those based on an average temperature threshold of the 90th percentile under different durations (2, 3, and 4 days) [14,15]. However, in our study, we found that the effects of the 93th percentile were stronger than those of the 95th percentile, which might have occurred because fewer heat wave days are found when using the 95th percentile.

Comparing our results with previous studies focused on the added effects of heat waves on mortality, we found that the effect magnitude of a heat wave varied depending on different studies [6,17,24]. For example, in a southern Chinese city, the excess risk mortality on cardiovascular disease was 20.0% (95% CI: 10.1%, 29.9%) when the heat wave was defined as ≥4 days and using the ≥95th percentile of temperature [14]. Another study conducted in Seoul showed that heat waves caused a 22.9% (95% CI: 9.9%, 37.4%) increase in cardiovascular mortality with the same definitions [15]. These results are slightly higher than our result, with a 14% (95% CI: 3%, 26%) increase in cardiovascular mortality in the identical definition. This difference may have arisen because the threshold temperature of the 95th percentile that we chose in Beijing was lower (28.3 °C) than that used for the southern Chinese city (31.9 °C). However, some studies in the United States have shown a much smaller result, with a 3.74% (95% CI: 2.29%, 5.22%) increase on total mortality due to the heat wave, when it was defined as ≥95th percentile and ≥2 consecutive days [17]. Another research group even showed a smaller and more insignificant effect of heat wave as 0.0% (95% CI: −3.8%, 4.0%) when using the same definition [19]. Both of the studies mentioned above showed a smaller increase in the mortality than that found in our results compared to the effect magnitude from the same definitions of heat waves. These studies paid attention to the effect of a heat wave on non-accidental mortality rather than the effect on cardiovascular mortality, which might be the reason for the difference from our results. Many studies have revealed that the adverse effects of a heat wave are strongly apparent in people who have cardiovascular diseases, especially coronary heart disease and cerebrovascular disease [25,26,27,28,29]. Exposure to heat waves might cause cardiovascular stress, which can lead to changes in blood pressure and vasoconstriction and eventually contribute to an increased cardiovascular mortality [30]. In addition, some studies have indicated that different regions have various effects [17,19,31]. Such differences might be caused by variations in housing constructions, activity patterns and the use of air conditioners [32,33].

This study revealed a cumulative added heat wave effect on cardiovascular mortality independent of the high temperature effects in Beijing (China). Moreover, by modeling the heat waves effect as a continuous function of duration [9], we avoided a random duration standard and allowed a direct evaluation of the duration at which such an added effect became obvious. We observed that the added effect tended to increase due to sustained heat when the waves lasted for 5 days or more among the total and older groups. Compared with prior studies, the longer minimum duration of HW definitions had greater mortality risks [6,32]. It is reasonable to assume that heat waves that have a longer duration have a greater added effect on daily mortality [17].

When stratified by age, the overall heat effects were larger for the older group (over 65 years of age) in the four HWs (HW7, HW8, HW11 and HW12). This finding was consistent with the results from other countries [6,24,34,35]. There are some explanations for the increased sensitivity, one of which was that the temperature regulation ability is reduced with ageing, including a reduced sweating process, less skin blood flow, and a reduced redistribution of blood flow [36,37,38]. We also examined the difference in mortality between females and males and found that the added effect was stronger in females than in males. Some studies showed that females are more vulnerable to heat wave-related death than males [5,39], which might be attributed to the physiological differences between genders [40]. However, as for the male and female groups in our study, the added effect was not very robust when the step function and quadratic spline were used, which had a wide confidence interval. However, there could be a higher risk for circulatory death in males, although the difference was not significant between genders [41,42]. The inconsistent effect modification needs to be further explored by extending the time period or collecting more detailed information at the individual level, such as nutrition, exercise and acclimatization to heat.

This study has three important strengths. To our knowledge, this is the first study to report the added effect of heat waves in different definitions that proposes a health risk-based metric to define cardiovascular mortality simultaneously in Beijing, China. Second, we model the added effect of heat waves as a continuous function of duration and thus allow a direct estimation of the duration at which the added effect becomes obvious. The final strength of this study is that by adjusting for the possible confounding effects of relative humidity and the main air pollutant PM_10_, we found that the results are robust after adjusting for PM_10_. This may be due to a higher PM_10_ concentration in China than in other countries and many studies have reported that PM_10_ can result in health problems, especially in the circulatory system [43,44,45].

Although our findings will make a contribution to the field, it is undeniable that our study has some limitations. First, by using a single city—Beijing—as our study area, our findings may not be generalized to other districts with various populations, climates, or housing structures. Further, we used the climate and pollutant data from fixed monitoring sites outside instead of a direct measurement indoors, which might have led to some estimated error. Moreover, although we adjusted the confounding factors of the relative humidity and PM_10_, we did not control for air conditioning and other pollutants because such data were not available. Finally, only total cardiovascular mortality data were used in this study, and future studies on cause-specific mortality are needed to examine the added effect.

## 5. Conclusions

This study suggests that heat waves have a significant added effect on cardiovascular mortality. We found that HWs are crucial for the relationship between heat waves and mortality, and we suggest that using HW8 with a daily average temperature ≥93th percentile and ≥5 consecutive days is the best definition in Beijing to capture the added effect on cardiovascular mortality. The effects of heat waves are modified by individual characteristics, such as age and gender. We observed that the added effect tends to increase due to sustained heat when the waves last for five days or more among the total and old groups. These findings may have implications for designing local heat warning systems and for protecting cardiovascular health during heat waves.

## Figures and Tables

**Table 1:** Definitions of heat waves and heat wave days in Beijing, 2006–2009.

Heat Wave	Definitions		*n*	Longest Duration (Days)
	Mean Temperature	Duration		
HW1	≥90th percentile (27.0 °C)	≥2 consecutive days	106	15
HW2	≥90th percentile (27.0 °C)	≥3 consecutive days	74	14
HW3	≥90th percentile (27.0 °C)	≥4 consecutive days	55	13
HW4	≥90th percentile (27.0 °C)	≥5 consecutive days	42	12
HW5	≥93th percentile (27.7 °C)	≥2 consecutive days	65	14
HW6	≥93th percentile (27.7 °C)	≥3 consecutive days	38	13
HW7	≥93th percentile (27.7 °C)	≥4 consecutive days	24	12
HW8	≥93th percentile (27.7 °C)	≥5 consecutive days	19	11
HW9	≥95th percentile (28.3 °C)	≥2 consecutive days	43	11
HW10	≥95th percentile (28.3 °C)	≥3 consecutive days	25	10
HW11	≥95th percentile (28.3 °C)	≥4 consecutive days	20	9
HW12	≥95th percentile (28.3 °C)	≥5 consecutive days	16	8

HW1–HW12 denotes 12 heat wave definitions.

**Table 2:** Summaries of the mortalities and meteorological and pollutant data in Beijing, 2006–2009.

Data	Min	P25	P50	Mean	P75	Max
Mortality (count)						
Total	14	39	46	47	54	85
Old (≥65 years)	13	32	38	39	45	75
Young (<65 years)	1	6	8	8	10	20
Female	6	17	21	21	25	42
Male	4	21	25	26	30	50
Meteorological index						
Mean temperature (°C)	−10.1	2.9	15	13.6	23.9	31.6
RH (%)	8	36	53	52.6	69	97
Air pollutants						
PM_10_ (µg/m^3^)	7	78	124	138.4	170	600

RH: relative humidity; PM_10_, particulate matter with an aerodynamic diameter of less than 10 μm in size.

**Table 3:** Relative risk of cardiovascular mortality due to the added effect of heat waves in Beijing under different heat wave definitions in the model, 2006–2009.

Heat Wave	RR (95% CI)				
	Total	Old	Young	Female	Male
HW1	1.02 (0.96, 1.09)	1.00 (0.93, 1.08)	1.12 (0.94, 1.34)	0.96 (0.86, 1.07)	1.08 (0.98, 1.18)
HW2	0.98 (0.91, 1.04)	0.97 (0.90, 1.04)	1.00 (0.84, 1.19)	0.91 (0.82, 1.01)	1.03 (0.94, 1.13)
HW3	1.01 (0.94, 1.08)	1.01 (0.94, 1.09)	0.96 (0.80, 1.15)	0.98 (0.88, 1.09)	1.04 (0.95, 1.14)
HW4	1.02 (0.95, 1.10)	1.02 (0.94, 1.11)	0.97 (0.80, 1.18)	1.01 (0.90, 1.14)	1.03 (0.93, 1.14)
HW5	1.03 (0.96, 1.11)	1.02 (0.94, 1.10)	1.12 (0.93, 1.35)	1.01 (0.90, 1.13)	1.05 (0.95, 1.16)
HW6	1.03 (0.95, 1.11)	1.01 (0.93, 1.10)	1.12 (0.92, 1.37)	1.05 (0.93, 1.19)	1.01 (0.90, 1.12)
HW7	1.10 (1.00, 1.21) *	1.14 (1.02, 1.27) *	0.93 (0.72, 1.21)	1.17 (1.00, 1.36) *	1.05 (0.91, 1.20)
HW8	1.18 (1.06, 1.31) *	1.24 (1.10, 1.39) *	0.96 (0.72, 1.28)	1.22 (1.03, 1.44) *	1.14 (0.98, 1.33)
HW9	1.00 (0.92, 1.08)	1.00 (0.91, 1.09)	1.00 (0.81, 1.24)	0.94 (0.82, 1.06)	1.05 (0.94, 1.18)
HW10	1.08 (0.98, 1.19)	1.09 (0.98, 1.21)	1.06 (0.83, 1.36)	1.08 (0.93, 1.25)	1.08 (0.94, 1.23)
HW11	1.14 (1.03, 1.26) *	1.18 (1.06, 1.32) *	0.97 (0.74, 1.26)	1.20 (1.03, 1.40) *	1.09 (0.95, 1.26)
HW12	1.14 (1.03, 1.27) *	1.21 (1.08, 1.36) *	0.87 (0.66, 1.16)	1.21 (1.03, 1.42) *	1.09 (0.94, 1.27)

Data are expressed as mean (95% confidence interval) and are controlled for seasonality, day of the week, relative humidity, temperature and PM_10_. RR: relative risk; CI: confidence interval; * *p* < 0.05.

## Supplementary Materials

The following are available online at www.mdpi.com/1660-4601/13/9/933/s1, Table S1: Sensitivity analysis on the degrees of freedom (*df*) for seasonality and relative humidity functions on the added effect under 12 different heat wave definitions in different age groups, Table S2: Sensitivity analysis on the degrees of freedom (*df*) for seasonality and relative humidity functions on the added effect under 12 different heat wave definitions in different gender, Table S3: Cumulative relative risk of the mortality due to the added wave effect in Beijing under different heat wave definitions in the model without adjusting PM_10_, 2006–2009, Figure S1: The residual autocorrelation figure of the model in the definition of ≥2 consecutive heat wave days above 93th percentile temperature, Figure S2: The residual autocorrelation figure of the model in the definition of ≥3 consecutive heat wave days above 93th percentile temperature, Figure S3: The residual autocorrelation figure of the model in the definition of ≥4 consecutive heat wave days above 93th percentile temperature, Figure S4: The relative risk figure of the circulatory mortality due to the added effect of heat waves in Beijing under different heat waves definitions in the model, 2006–2009.
