# Supplementary material for: Impact of Heat Wave Definitions on the Added Effect of Heat Waves on Cardiovascular Mortality in Beijing, China
Source: Int J Environ Res Public Health. 2016 Sep 21;13(9):933. doi: 10.3390/ijerph13090933 (PMC5036765; doi:10.3390/ijerph13090933)
Supplement: Supplementary file 1 [file ijerph-13-00933-s001.pdf]

Supplementary Materials: Impact of Heat Wave Definitions to the Added Effect of Heat Waves on Cardiovascular Mortality in Beijing, China

Wentan Dong, Qiang Zeng, Yue Ma, Guoxing Li and Xiaochuan Pan

Table S1. Sensitivity analysis on the degrees of freedom (*df*) for seasonality and relative humidity functions on the added effect under 12 different heat wave definitions in different age groups.

	Heat Wave	Seasonality (*df*)		Relative Humidity (*df*)	
		2	4	2	4
Total	HW1	1.03 (0.96, 1.10)	1.02 (0.96, 1.09)	1.02 (0.96, 1.09)	1.02 (0.96, 1.10)
	HW2	0.97 (0.91, 1.04)	0.98 (0.91, 1.04)	0.98 (0.91, 1.04)	0.97 (0.91, 1.04)
	HW3	1.01 (0.94, 1.08)	1.01 (0.94, 1.08)	1.01 (0.94, 1.08)	1.01 (0.94, 1.08)
	HW4	1.02 (0.95, 1.10)	1.02 (0.95, 1.10)	1.02 (0.95, 1.10)	1.02 (0.95, 1.10)
	HW5	1.03 (0.96, 1.11)	1.03 (0.96, 1.11)	1.03 (0.96, 1.11)	1.03 (0.96, 1.11)
	HW6	1.02 (0.94, 1.10)	1.03 (0.95, 1.11)	1.03 (0.95, 1.11)	1.02 (0.95, 1.11)
	HW7	1.09 (0.98, 1.20) *	1.10 (0.99, 1.21)	1.10 (0.99, 1.21)	1.09 (0.99, 1.21)
	HW8	1.17 (1.05, 1.30) *	1.18 (1.06, 1.31) *	1.17 (1.06, 1.31) *	1.17 (1.05, 1.30) *
	HW9	0.99 (0.91, 1.07)	0.99 (0.92, 1.08)	0.99 (0.92, 1.08)	0.99 (0.91, 1.08)
	HW10	1.06 (0.97, 1.17)	1.08 (0.98, 1.18)	1.07 (0.98, 1.18)	1.07 (0.97, 1.18)
	HW11	1.13 (1.02, 1.24) *	1.14 (1.03, 1.26) *	1.14 (1.03, 1.26) *	1.13 (1.02, 1.25) *
	HW12	1.13 (1.02, 1.26) *	1.14 (1.03, 1.27) *	1.14 (1.03, 1.26) *	1.14 (1.03, 1.26) *
Old	HW1	1.00 (0.93, 1.08)	1.00 (0.93, 1.08)	1.00 (0.93, 1.08)	1.00 (0.93, 1.08)
	HW2	0.97 (0.90, 1.04)	0.97 (0.90, 1.04)	0.97 (0.90, 1.04)	0.97 (0.90, 1.04)
	HW3	1.01 (0.93, 1.09)	1.01 (0.94, 1.09)	1.01 (0.94, 1.09)	1.01 (0.93, 1.09)
	HW4	1.02 (0.94, 1.11)	1.02 (0.94, 1.11)	1.02 (0.94, 1.11)	1.02 (0.94, 1.11)
	HW5	1.02 (0.94, 1.10)	1.02 (0.94, 1.10)	1.02 (0.94, 1.10)	1.01 (0.93, 1.10)
	HW6	1.00 (0.92, 1.09)	1.01 (0.93, 1.10)	1.01 (0.93, 1.10)	1.01 (0.92, 1.10)
	HW7	1.13 (1.01, 1.25) *	1.14 (1.02, 1.27) *	1.14 (1.02, 1.27) *	1.13 (1.02, 1.26) *
	HW8	1.22 (1.09, 1.37) *	1.24 (1.10, 1.39) *	1.23 (1.10, 1.39) *	1.23 (1.09, 1.38) *
	HW9	0.98 (0.90, 1.08)	1.00 (0.91, 1.09)	0.99 (0.91, 1.09)	0.99 (0.90, 1.08)
	HW10	1.07 (0.97, 1.19)	1.09 (0.98, 1.21)	1.09 (0.98, 1.21)	1.08 (0.97, 1.20)
	HW11	1.16 (1.04, 1.30) *	1.18 (1.06, 1.32) *	1.18 (1.05, 1.31) *	1.17 (1.05, 1.31) *
	HW12	1.20 (1.07, 1.34) *	1.21 (1.08, 1.36) *	1.21 (1.08, 1.35) *	1.20 (1.07, 1.35) *
Young	HW1	1.12 (0.94, 1.34)	1.12 (0.94, 1.34)	1.12 (0.94, 1.34)	1.12 (0.93, 1.33)
	HW2	1.00 (0.84, 1.19)	1.00 (0.84, 1.19)	1.00 (0.84, 1.19)	1.01 (0.85, 1.20)
	HW3	0.96 (0.80, 1.15)	0.96 (0.81, 1.15)	0.96 (0.80, 1.15)	0.97 (0.81, 1.16)
	HW4	0.97 (0.80, 1.18)	0.98 (0.80, 1.18)	0.98 (0.80, 1.18)	0.97 (0.80, 1.18)
	HW5	1.12 (0.93, 1.35)	1.11 (0.92, 1.34)	1.11 (0.92, 1.34)	1.13 (0.94, 1.36)
	HW6	1.12 (0.92, 1.37)	1.12 (0.92, 1.37)	1.12 (0.92, 1.37)	1.13 (0.93, 1.38)
	HW7	0.93 (0.72, 1.21)	0.93 (0.72, 1.20)	0.93 (0.72, 1.20)	0.95 (0.73, 1.23)
	HW8	0.97 (0.73, 1.28)	0.96 (0.72, 1.28)	0.96 (0.72, 1.28)	0.98 (0.74, 1.31)
	HW9	1.00 (0.81, 1.23)	0.99 (0.80, 1.23)	0.99 (0.80, 1.23)	1.01 (0.82, 1.26)
	HW10	1.06 (0.83, 1.35)	1.05 (0.82, 1.35)	1.05 (0.82, 1.34)	1.09 (0.85, 1.41)
	HW11	0.97 (0.75, 1.26)	0.96 (0.74, 1.25)	0.96 (0.74, 1.26)	0.99 (0.76, 1.29)
	HW12	0.88 (0.66, 1.16)	0.87 (0.66, 1.15)	0.87 (0.66, 1.15)	0.89 (0.67, 1.18)

Table S2. Sensitivity analysis on the degrees of freedom (*df*) for seasonality and relative humidity functions on the added effect under 12 different heat wave definitions in different gender.

	Heat Wave	Seasonality (*df*)		Relative Humidity (*df*)	
		2	4	2	4
Female	HW1	0.96 (0.86, 1.07)	0.96 (0.86, 1.07)	0.96 (0.87, 1.07)	0.96 (0.87, 1.07)
	HW2	0.91 (0.82, 1.01)	0.91 (0.82, 1.01)	0.91 (0.82, 1.01)	0.91 (0.82, 1.00)
	HW3	0.98 (0.88, 1.09)	0.97 (0.87, 1.08)	0.98 (0.88, 1.09)	0.97 (0.87, 1.08)
	HW4	1.02 (0.91, 1.14)	1.01 (0.90, 1.14)	1.02 (0.91, 1.14)	1.01 (0.90, 1.14)

	HW5	1.01 (0.90, 1.13)	1.02 (0.91, 1.14)	1.01 (0.90, 1.13)	1.01 (0.90, 1.13)
	HW6	1.05 (0.93, 1.19)	1.05 (0.93, 1.19)	1.05 (0.93, 1.18)	1.04 (0.92, 1.18)
	HW7	1.17 (1.00, 1.36) *	1.17 (1.00, 1.36) *	1.16 (0.99, 1.35)	1.15 (0.99, 1.35)
	HW8	1.22 (1.03, 1.44) *	1.22 (1.04, 1.45) *	1.21 (1.03, 1.43) *	1.21 (1.02, 1.42) *
	HW9	0.94 (0.83, 1.07)	0.94 (0.83, 1.07)	0.93 (0.82, 1.06)	0.93 (0.81, 1.05)
	HW10	1.08 (0.93, 1.25)	1.09 (0.94, 1.27)	1.06 (0.92, 1.23)	1.06 (0.91, 1.23)
	HW11	1.20 (1.03, 1.40) *	1.21 (1.04, 1.42) *	1.19 (1.02, 1.39) *	1.19 (1.01, 1.39) *
	HW12	1.21 (1.03, 1.42) *	1.22 (1.04, 1.43) *	1.20 (1.02, 1.41) *	1.20 (1.02, 1.41) *
Male	HW1	1.08 (0.99, 1.19)	1.08 (0.98, 1.18)	1.08 (0.98, 1.18)	1.08 (0.98, 1.18)
	HW2	1.03 (0.94, 1.13)	1.03 (0.94, 1.13)	1.03 (0.94, 1.13)	1.03 (0.94, 1.13)
	HW3	1.03 (0.94, 1.14)	1.04 (0.95, 1.15)	1.04 (0.95, 1.14)	1.04 (0.94, 1.14)
	HW4	1.02 (0.92, 1.13)	1.03 (0.93, 1.14)	1.03 (0.93, 1.14)	1.03 (0.93, 1.14)
	HW5	1.05 (0.95, 1.16)	1.04 (0.94, 1.15)	1.05 (0.95, 1.16)	1.05 (0.95, 1.16)
	HW6	0.99 (0.89, 1.11)	1.00 (0.90, 1.12)	1.01 (0.90, 1.12)	1.01 (0.90, 1.12)
	HW7	1.02 (0.89, 1.17)	1.04 (0.91, 1.20)	1.05 (0.91, 1.20)	1.04 (0.91, 1.20)
	HW8	1.12 (0.96, 1.31)	1.14 (0.98, 1.32)	1.14 (0.98, 1.33)	1.14 (0.98, 1.33)
	HW9	1.03 (0.92, 1.15)	1.04 (0.93, 1.17)	1.05 (0.94, 1.18)	1.05 (0.93, 1.18)
	HW10	1.05 (0.91, 1.20)	1.06 (0.93, 1.21)	1.08 (0.95, 1.23)	1.07 (0.94, 1.23)
	HW11	1.07 (0.92, 1.23)	1.08 (0.94, 1.24)	1.10 (0.95, 1.26)	1.09 (0.94, 1.26)
	HW12	1.07 (0.92, 1.24)	1.09 (0.94, 1.26)	1.10 (0.95, 1.27)	1.09 (0.94, 1.27)

Data are mean (95% confidence interval) and are controlled for seasonality, day of the week, relative humidity, temperature and PM_{10}. PM_{10}, particulate matter with an aerodynamic diameter of less than 10 μm. RR relative risk, 95% CI: 95% confidence interval, * $p < 0.05$.

Table S3. Cumulative relative risk of the mortality due to the added wave effect in Beijing under different heat wave definitions in the model without adjusting PM_{10}, 2006–2009.

Heat Wave	RR (95% CI)				
	Total	Old	Young	Female	Male
HW1	1.02 (0.96, 1.09)	1.00 (0.93, 1.07)	1.13 (0.95, 1.35)	0.96 (0.87, 1.07)	1.07 (0.98, 1.18)
HW2	0.97 (0.91, 1.04)	0.97 (0.90, 1.04)	1.00 (0.84, 1.18)	0.92 (0.83, 1.01)	1.02 (0.93, 1.12)
HW3	1.01 (0.95, 1.08)	1.01 (0.94, 1.09)	0.96 (0.81, 1.15)	0.97 (0.88, 1.08)	1.05 (0.95, 1.15)
HW4	1.03 (0.96, 1.10)	1.02 (0.94, 1.11)	0.99 (0.82, 1.20)	1.01 (0.91, 1.13)	1.04 (0.94, 1.15)
HW5	1.04 (0.97, 1.12)	1.02 (0.94, 1.10)	1.13 (0.94, 1.36)	1.02 (0.92, 1.14)	1.05 (0.95, 1.16)
HW6	1.03 (0.95, 1.11)	1.01 (0.93, 1.10)	1.14 (0.93, 1.38)	1.04 (0.93, 1.18)	1.02 (0.91, 1.13)
HW7	1.11 (1.00, 1.22) *	1.14 (1.03, 1.27) *	0.96 (0.74, 1.23)	1.16 (1.00, 1.34) *	1.06 (0.93, 1.22)
HW8	1.18 (1.06, 1.31) *	1.23 (1.10, 1.38) *	0.98 (0.74, 1.30)	1.21 (1.03, 1.42) *	1.16 (1.00, 1.34) *
HW9	1.00 (0.92, 1.08)	1.00 (0.91, 1.09)	1.01 (0.82, 1.25)	0.93 (0.82, 1.05)	1.06 (0.95, 1.19)
HW10	1.08 (0.98, 1.19)	1.09 (0.98, 1.20)	1.08 (0.85, 1.37)	1.07 (0.92, 1.23)	1.09 (0.95, 1.24)
HW11	1.14 (1.04, 1.26) *	1.18 (1.06, 1.31) *	0.99 (0.77, 1.28)	1.19 (1.02, 1.38) *	1.11 (0.96, 1.27)
HW12	1.15 (1.04, 1.27) *	1.21 (1.09, 1.36) *	0.89 (0.67, 1.17)	1.20 (1.03, 1.41) *	1.11 (0.96, 1.28)

Data are mean (95% confidence interval) and are controlled for seasonality, day of the week, relative humidity, temperature. RR relative risk, 95% CI: 95% confidence interval, * $p < 0.05$.

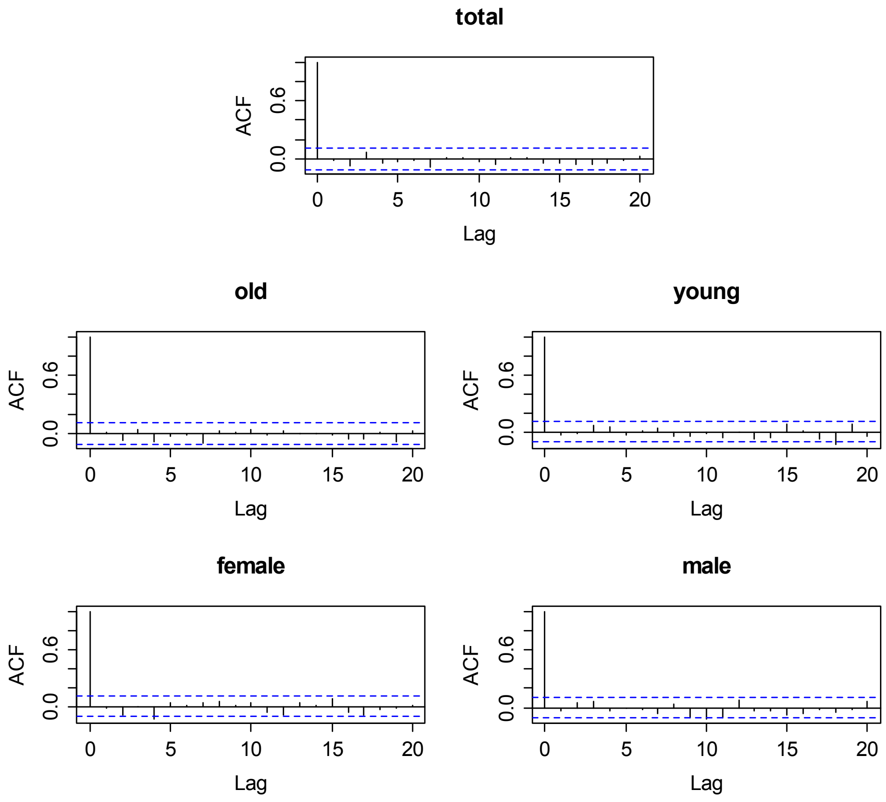

Figure S1. The residual autocorrelation figure of the model in the definition of ≥2 consecutive heat wave days above 93th percentile temperature.

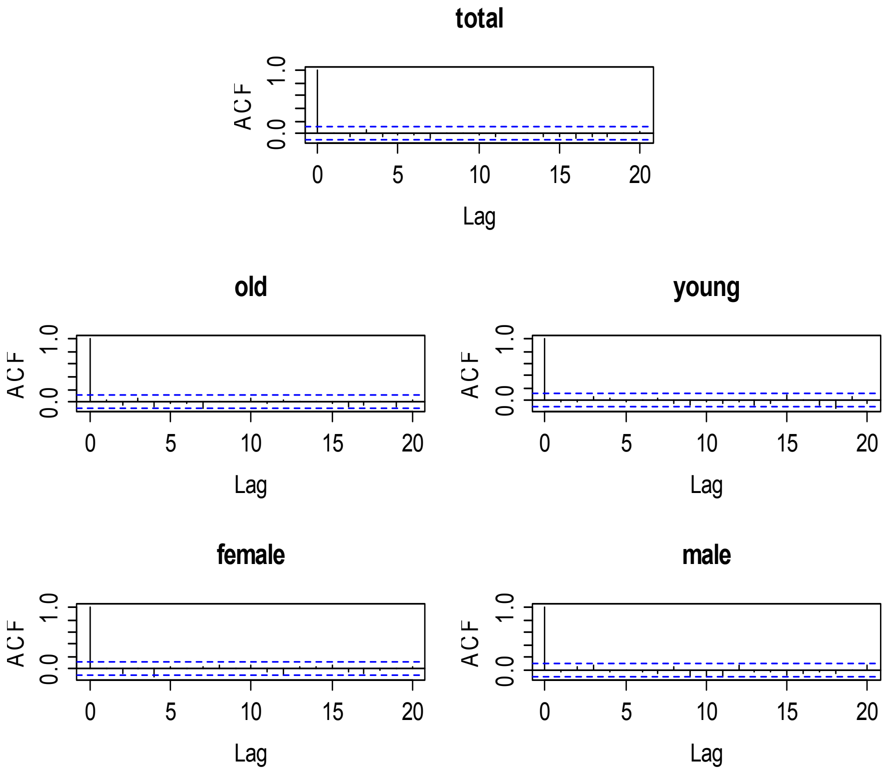

Figure S2. The residual autocorrelation figure of the model in the definition of ≥3 consecutive heat wave days above 93th percentile temperature.

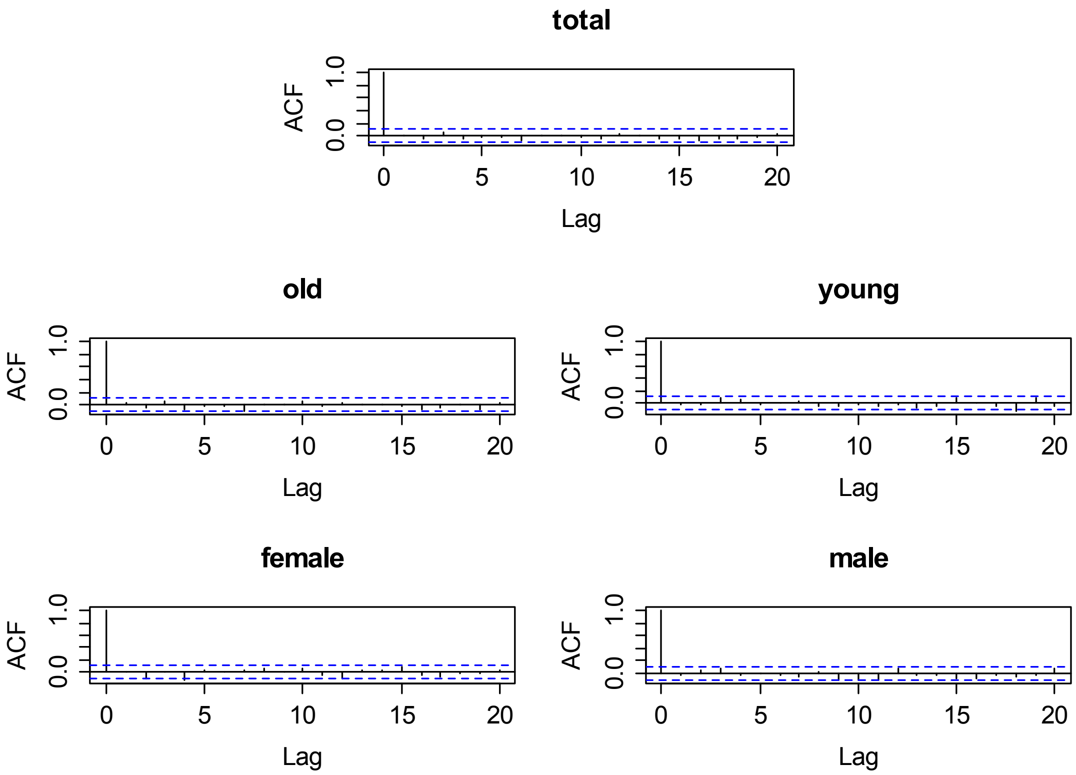

Figure S3. The residual autocorrelation figure of the model in the definition of ≥4 consecutive heat wave days above 93th percentile temperature.

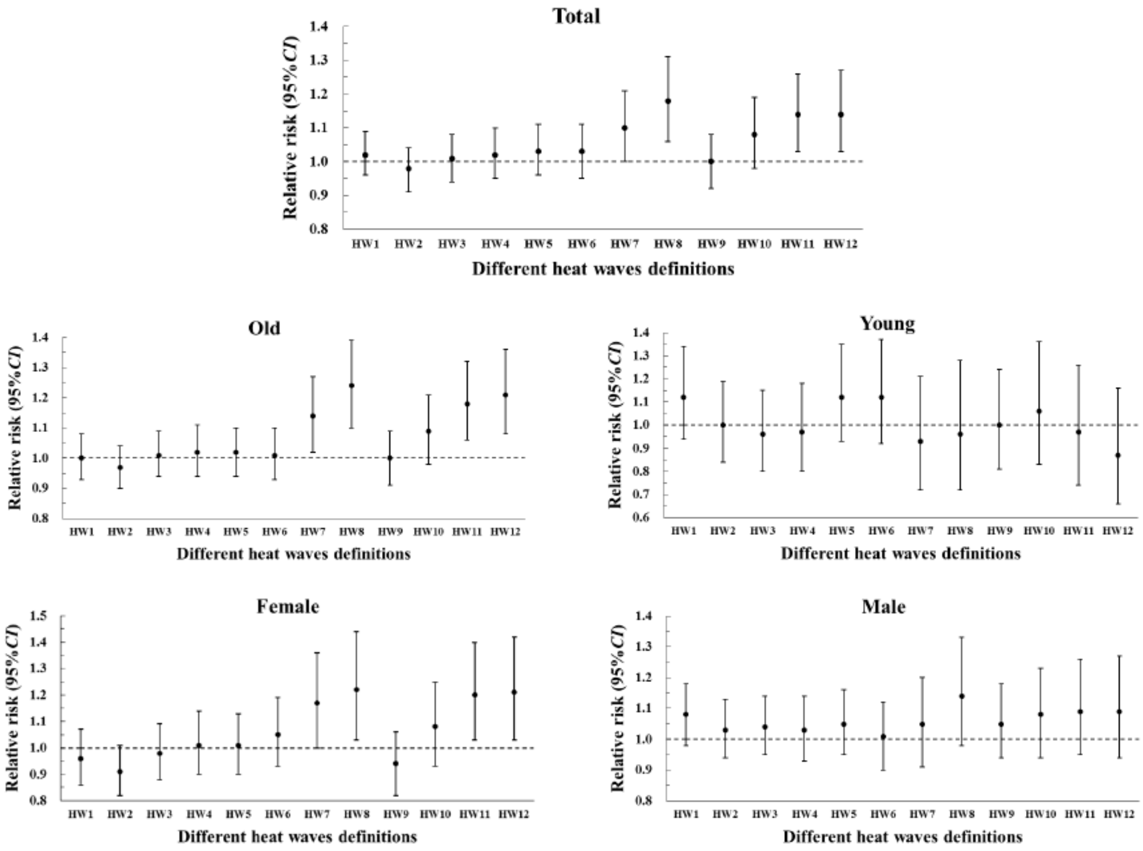

Figure S4. The relative risk figure of the circulatory mortality due to the added effect of heat waves in Beijing under different heat waves definitions in the model, 2006–2009.
